# An abluminal biodegradable polymer sirolimus-eluting stent versus a durable polymer everolimus-eluting stent in patients undergoing coronary revascularization: 3-year clinical outcomes of a randomized non-inferiority trial

**DOI:** 10.1038/s41598-019-54964-8

**Published:** 2019-12-06

**Authors:** Haijun Zhang, Xiaoping Zhang, Yuxia Yin, Chao Zhou, Wei Deng, Junwei Zhang, Wenbo Hou, Shoutao Lu, Caixia Song, Xiaoshan Cui, Shenguo Wang, Fei Yang, Guang Liu, Cuihai Duan, Junbo Ge

**Affiliations:** 1 Intervention & Vascular Surgery, Medical College of Tongji University, Shanghai, China; 2 Department of Health Science and Technology, Faculty of Medicine, Aalborg University, Aalborg Ø, Denmark; 3 National Joint Engineering Laboratory for Biomedical Material Modification, Shandong, China; 4 Key Laboratory of Public Health Safety, Ministry of Education, School of Public Health, Fudan University, Shanghai, China; 5 Institute of Chemistry, Chinese Academy of Sciences, Beijing, China; 6 Shanghai Institute of Cardiovascular Diseases, Zhongshan Hospital, Fudan University, Shanghai, China

**Keywords:** Interventional cardiology, Ischaemia

## Abstract

The Cordimax stent has proved non-inferior to the Cypher Select durable polymer sirolimus-eluting stent for the primary endpoint of angiographic in-stent late luminal loss and in-stent mean diameter stenosis at 9 months. The trial was designed to compare the efficacy and safety of the Cordimax stent with the Xience V stent in patients undergoing coronary revascularization. This randomized, multicenter trial enrolled 3697 patients treated with Cordimax stent (2460 patients) and Xience V stent (1237 patients). The primary efficacy endpoint was a target-lesion failure (TLF) at 1 year and the primary safety endpoint was a composite of death or myocardial infarction (MI) at 3 years. 3399 patients (91.9%) completed 3-year follow-up. At 1 year, the primary efficacy endpoint occurred in 86 (3.5%) patients in the Cordimax group versus 40 (3.2%) patients in the Xience V group (0.3% absolute risk difference, 95% CI −1.0–1.5%, *P*_non-inferiority_ < 0.0001). At 3 years, the primary safety endpoint occurred in 39 (1.6%) patients in the Cordimax group versus 19 (1.5%) patients in the Xience V group (0.05% absolute risk difference, 95% CI −0.8–0.9%, *P*_non-inferiority_ < 0.0001). The incidence of target lesion revascularization was low in Cordimax group compared with Xience V group (3.6% versus 5.1%, *P* = 0.03). There were no differences between Cordimax and Xience V in terms of Cardiac death (0.3% versus 0.4%, *P* = 0.70), myocardial infarction (1.2% versus 0.9%, *P* = 0.37), and the stent thrombosis (0.4% versus 0.6%, *P* = 0.61). In conclusion, **s**afety and efficacy outcomes of Cordimax stent were non-inferior to the Xience V stent 3 years after stent implantation.

## Introduction

Drug-eluting stents (DES) with controlled release of antiproliferative agents from a durable polymer have effectively reduced the incidence of restenosis and major cardiac events including myocardial infarction (MI) and cardiac death compared with bare-metal stents (BMS)^1,2^. However, first generation durable polymer DES have been linked to increased risk of stent thrombosis (ST), especially very late ST (VLST) after discontinuation of dual antiplatelet therapy^3,4^. The durable polymer matrix has been implicated as a likely trigger of delayed reendothelialization and chronic inflammation leading to these late complications^5,6^. The introduction of newer DES of improved durable polymers or biodegradable polymers has significantly reduced the risk of cardiac complications after stent implantation^7^. Cordimax (Rientech Medical, Shandong, China) is a novel biodegradable polymer sirolimus-eluting stent with an asymmetric coating, eluting the drug solely to the abluminal surface. Compared with a stent with conventional coating, Cordimax has demonstrated favorable drug release profile *in vitro* and *in vivo*^8^ and afforded enhanced endothelialization and vascular healing after implantation *in vivo*^9^. In our previous randomized clinical trial comparing the safety and efficacy of Cordimax and Cypher Select (Cordis^®^, Miami Lakes, FL, USA), a durable polymer sirolimus-eluting stent, Cordimax demonstrated non-inferiority to Cypher Select at 9-month angiographic and 1-year clinical follow-up^10^. Cypher was the first-generation drug eluting stent (DES) which has a non-degradable coating and is made of 316 L stainless steel, which suppresses the occurrence of restenosis associated with bare metal stents (BMS)^11,12^. However, due to the high incidence of very late stent thrombosis its secure usage had been challenged^13,14^. Xience V (Abbott Vascular, Santa Clara, CA, USA) is the newer generation DES which is made of thinner stent and its safety and effectiveness has been demonstrated in multiple clinical trials^15^. The incidence of thrombosis using Xience V had been observed to be significantly lower than the Cypher^16^. Therefore, the aim of this clinical trial was to evaluate the safety and efficacy of Cordimax compared with Xience V, a durable polymer everolimus-eluting stent, at 3-year clinical follow-up.

## Results

**Figure 1:**
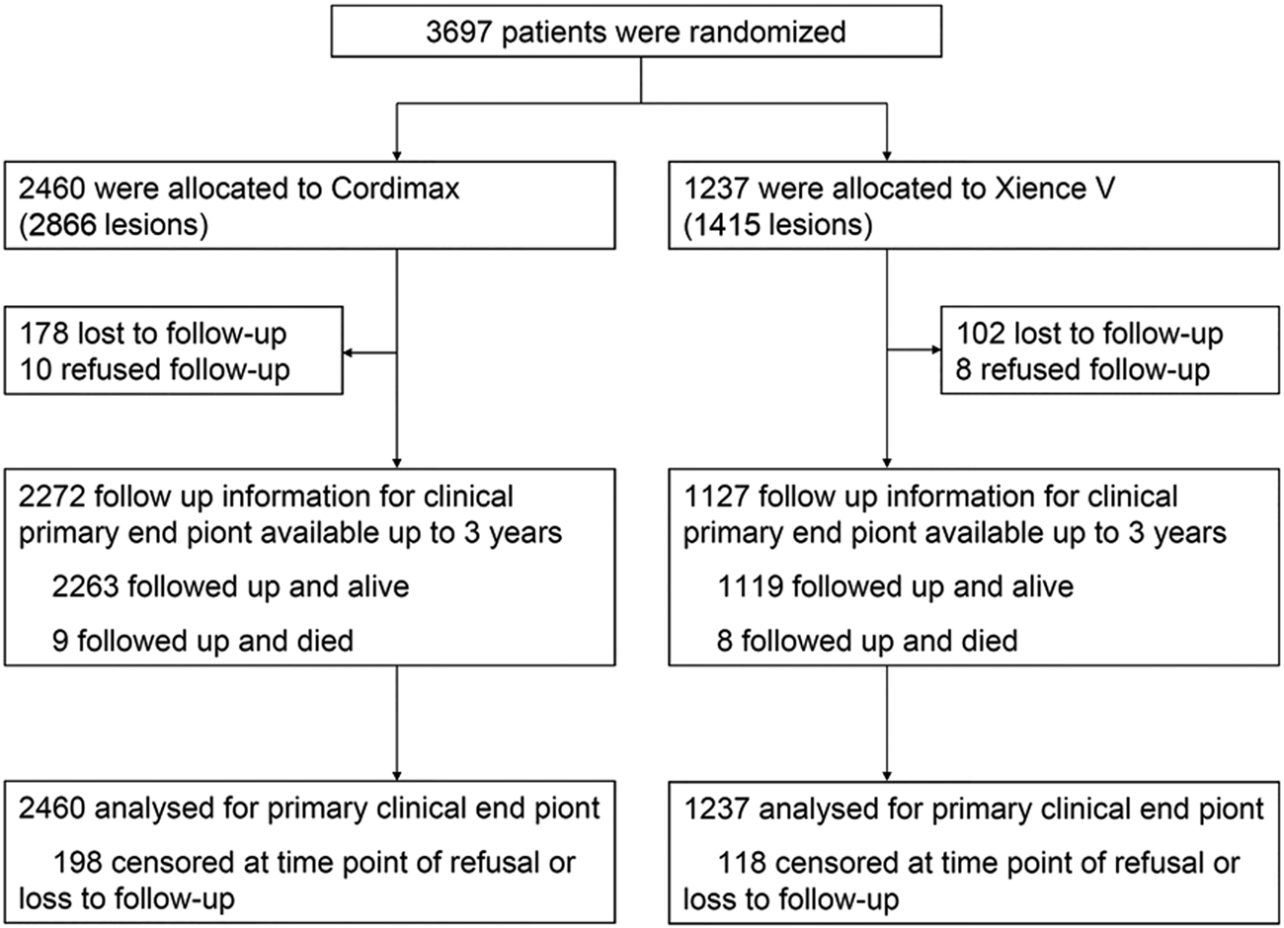
Trial profile. Enrollment and Randomization of Patients Who underwent coronary revascularization.

A total of 3697 patients with 4281 lesions were randomly assigned to receive either the Cordimax biodegradable polymer sirolimus-eluting stent (2460 patients with 2866 lesions) or the Xience V durable polymer everolimus-eluting stent (1237 patients with 1415 lesions; Fig. 1) in a 2:1 allocation. The baseline demographics and the clinical and angiographic characteristics of patients in the two treatment arms were well balanced except for CCS (Canadian Cardiovascular Society)/Braundward angina pectoris class, and NYHA (New York Heart Association) functional class (Table 1). Higher proportions of patients in the Cordimax arm had angina class IV (10.7% of Cordimax vs. 6.9% of Xience V) and NYHA class III or IV (21.9% and 1.5% of Cordimax vs. 14.8% and 0.6% of Xience V) (Table 1). The total stent length per patient was slightly shorter in the Cordimax group (25.00 mm Cordimax vs. 25.99 mm Xience V, *P* = 0.20; Table 1). A total of 3399 patients (91.9%; 2272 in the Cordimax arm and 1127 in the Xience V arm) completed 3-year follow-up (Fig. 1).

**Table 1:** Baseline Patient and Lesion Characteristics.

Characteristic	Cordimax	Xience V	*P* value
Number of patients	2460	1237	
Age, y	60.74 ± 10.74	60.08 ± 10.67	0.39
Male gender	1749 (71.1%)	923 (74.6%)	0.02
Cardiovascular risk factors			
Diabetes mellitus	480 (19.5%)	240 (19.4%)	0.94
Hypertension	1369 (55.7%)	741 (59.9%)	0.01
Current smoker	870 (35.4%)	451 (36.5%)	0.51
Past medical history			
Previous MI	385 (15.7%)	143 (11.6%)	0.001
Previous Stroke	86 (3.5%)	23 (1.9%)	0.01
Previous PCI	197 (8.0%)	76 (6.1%)	0.04
Previous CABG	12 (0.5%)	4 (0.3%)	0.47
CCS/Braunwald			< 0.0001
I	331 (13.5%)	200 (16.2%)	
II	994 (40.4%)	562 (45.4%)	
III	851 (34.6%)	384 (31.0%)	
IV	263 (10.7%)	85 (6.9%)	
NYHA			<0.0001
I	876 (35.6%)	454 (36.7%)	
II	982 (39.9%)	581 (47.0%)	
III	538 (21.9%)	183 (14.8%)	
IV	38 (1.5%)	8 (0.6%)	
Number of lesions	2866	1415	
Treated lesions per patient	1.17 ± 0.46	1.14 ± 0.41	0.50
Stents per patient	1.26 ± 0.55	1.22 ± 0.50	0.39
Stent length per patient, mm	25.00 ± 10.78	25.99 ± 10.68	0.20
Location of target lesion			0.04
Left anterior descending coronary	1543 (62.7%)	761 (61.5%)	
Left circumflex coronary artery	490 (19.9%)	286 (23.1%)	
Right coronary artery	768 (31.2%)	337 (27.2%)	
Left main artery	65 (2.6%)	31 (2.5%)	

Values are presented as mean ± SD or n (%) for categorical variables.

**Figure 2:**
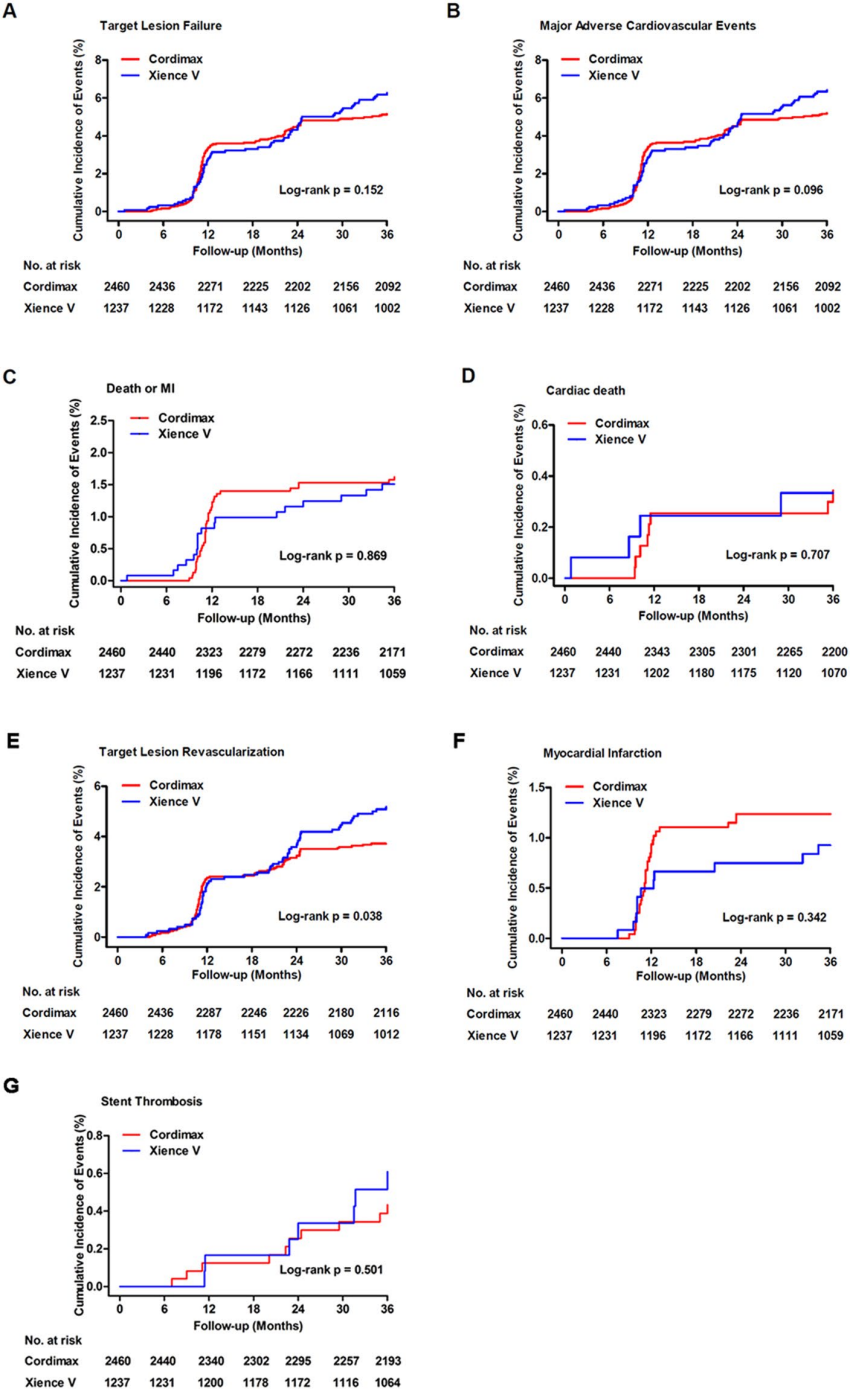
Kaplan-Meier curves. Kaplan-Meier curves for the primary endpoint (**A**) and major adverse cardiovascular events (**B**), the primary safety endpoint (**C**), cardiac death (**D**), target-lesion revascularization (**E**), myocardial infarction (**F**), and stent thrombosis (**G**) at 3 years. Primary endpoint was a composite of cardiac death, target-vessel MI, or clinically indicated target-lesion revascularization (CI-TLR). The primary safety endpoint was a composite of death or myocardial infarction.

At 1 year, the primary efficacy endpoint (TLF) occurred in 86 (3.5%) patients in the Cordimax group versus 40 (3.2%) patients in the Xience V group (Table 2, Fig. 2). With an absolute risk difference of 0.3% and the upper limit of the two-sided 95% CI at 1.5% (95% CI −1.0–1.5%), the Cordimax stent proved to be non-inferior to the Xience V stent at 1 year after implantation (*P*_non-inferiority_ < 0.0001). At 3 years, the primary efficacy endpoint (TLF) occurred in 121 (4.9%) patients in the Cordimax group versus 78 (6.3%) patients in the Xience V group (Table 2, Fig. 2). Non-inferiority of the Cordimax stent at 3 years was established with an absolute risk difference of −1.39% and the upper limit of the two-sided 95% CI at 0.2% (95% CI −3.0–0.2%, *P*_non-inferiority_ = 0.0242). With regard to the secondary clinical safety and efficacy endpoints, target lesion revascularization (3.6% versus 5.1%, *P* = 0.03) were less frequent among Cordimax-treated patients compared with Xience V-treated patients. There were no differences between Cordimax and Xience V in terms of Cardiac death (0.3% versus 0.4%, *P* = 0.70), myocardial infarction (1.2% versus 0.9%, *P* = 0.37), and the definite or probable stent thrombosis (0.4% versus 0.6%, *P* = 0.61) at 3 years. At 3 years, the primary safety endpoint occurred in 39 (1.6%) patients in the Cordimax group versus 19 (1.5%) patients in the Xience V group (Table 2, Fig. 2). With an absolute risk difference of 0.05% and the upper limit of the two-sided 95% CI at 0.9% (95% CI −0.8–0.9%, *P*_non-inferiority_ < 0.0001) demonstrating noninferiority of Cordimax stent relative to Xience V stent.

**Table 2:** Cumulative Clinical Events.

Events	Cordimax (n = 2460)	Xience V (n = 1237)	RR (95% CI)	*P* value
Events at 6 month				
TLF	4 (0.2%)	4 (0.3%)	0.50 (0.13–2.01)	0.32
All cause death	0 (0%)	1 (0.1%)	—	—
Cardiac death	0 (0%)	1 (0.1%)	—	—
MI	0 (0%)	0 (0%)	—	—
TLR (clinically driven)	4 (0.2%)	3 (0.2%)	0.67 (0.15–2.99)	0.60
Death or MI	0 (0%)	1 (0.1%)	—	—
MACE	4 (0.2%)	4 (0.3%)	0.50 (0.13–2.01)	0.32
Events at 1 year				
TLF	86 (3.5%)	40 (3.2%)	1.07 (0.74–1.55)	0.73
All cause death	7 (0.3%)	4 (0.3%)	0.88 (0.26–3.00)	0.84
Cardiac death	6 (0.2%)	3 (0.2%)	1.01 (0.25–4.01)	0.99
MI	27 (1.1%)	8 (0.6%)	1.70 (0.77–3.72)	0.18
TLR (clinically driven)	57 (2.3%)	28 (2.3%)	1.02 (0.66–1.60)	0.92
Death or MI	34 (1.4%)	12 (1.0%)	1.43 (0.74–2.74)	0.29
MACE	86 (3.5%)	40 (3.2%)	1.08 (0.75–1.56)	0.68
Events at 2 year				
TLF	113 (4.6%)	61 (4.9%)	0.93 (0.69–1.26)	0.65
All cause death	7 (0.3%)	6 (0.5%)	0.59 (0.20–1.74)	0.33
Cardiac death	6 (0.2%)	3 (0.2%)	1.01 (0.25–4.01)	0.99
MI	30 (1.2%)	9 (0.7%)	1.68 (0.80–3.52)	0.17
TLR (clinically driven)	82 (3.3%)	49 (4.0%)	0.84 (0.60–1.19)	0.33
Death or MI	37 (1.5%)	115 (1.2%)	1.24 (0.68–2.25)	0.48
MACE	114 (4.6%)	62 (5.0%)	0.93 (0.68–1.25)	0.61
Events at 3 year				
TLF	121 (4.9%)	78 (6.3%)	0.78 (0.59–1.03)	0.08
All cause death	9 (0.4%)	8 (0.6%)	0.57 (0.22–1.46)	0.23
Cardiac death	8 (0.3%)	5 (0.4%)	0.81 (0.26–2.45)	0.70
MI	30 (1.2%)	11 (0.9%)	1.37 (0.69–2.73)	0.37
TLR (clinically driven)	88 (3.6%)	63 (5.1%)	0.70 (0.51–0.96)	0.03
Death or MI	39 (1.6%)	19 (1.5%)	1.03 (0.60–1.78)	0.91
MACE	122 (5.0%)	79 (6.4%)	0.78 (0.59–1.02)	0.07

Values are n (%). *Relative risk (RR) and p values are from the chi-square test. MACE, Major Adverse Cardiovascular Event; MI, Myocardial Infarction; TLF, Target Lesion Failure; TLR, Target Lesion Revascularisation; and ST, Stent Thrombosis.

Between 1 and 2 years, the rates of the primary efficacy endpoint and the primary safety endpoint were similar in both groups. Rates of the combined and other individual components of the secondary endpoint between 1 and 2 years did not differ significantly between the two stent groups (Table 3). Between 2 and 3 years, lower portions of patients in the Cordimax group had TLF (0.3% versus 1.4%, P < 0.001), TLR (0.2% versus 1.1%, *P* < 0.001), and MACE (0.3% versus 1.4%, *P* < 0.001). The primary safety endpoint, and other individual components of the secondary endpoints did not differ significantly between the two stent groups (Table 4). At 3 years, the cumulative incidence of Definite or Probable Stent Thrombosis was not different between the 2 groups (0.4% versus 0.6%, *P* = 0.61; Table 5).

**Table 3:** Outcome differences between 1 year and 2 year.

Events	Cordimax (n = 2460)	Xience V (n = 1237)	HR (95% CI)	*P* value
TLF	28 (1.1%)	21 (1.7%)	0.67 (0.38–1.18)	0.16
All cause death	0 (0%)	2 (0.2%)	—	—
Cardiac death	0 (0%)	0 (0%)	—	—
MI	3 (0.2%)	1 (0.1%)	1.51 (0.16–14.49)	0.72
TLR (clinically driven)	25 (1.0%)	21 (1.7%)	1.01 (1.00–1.07)	0.08
Death or MI	3 (0.2%)	3 (0.2%)	0.50 (0.10–2.49)	0.39
MACE	28 (1.1%)	22 (1.8%)	0.64 (0.37–1.11)	0.11
ST	4 (0.2%)	2 (0.2%)	1.01 (0.18–5.48)	1.00

Values are n (%). *Relative risk (RR) and p values are from the chi-square test. MACE, Major Adverse Cardiovascular Event; MI, Myocardial Infarction; TLF, Target Lesion Failure; TLR, Target Lesion Revascularisation; and ST, Stent Thrombosis.

**Table 4:** Outcome differences between 2 year and 3 year.

Events	Cordimax	Xience V	RR (95% CI)	*P* value
	(n = 2460)	(n = 1237)		
TLF	8 (0.3%)	17 (1.4%)	0.24 (0.10–0.55)	<0.001
All cause death	2 (0.1%)	2 (0.2%)	0.50 (0.07–3.57)	0.48
Cardiac death	2 (0.1%)	2 (0.2%)	0.50 (0.07–3.57)	0.48
MI	0 (0%)	2 (0.2%)	—	—
TLR (clinically driven)	6 (0.2%)	14 (1.1%)	0.22 (0.08–0.56)	<0.001
Death or MI	2 (0.1%)	4 (0.3%)	0.25 (0.05–1.37)	0.08
MACE	8 (0.3%)	17 (1.4%)	0.24 (0.10–0.55)	<0.001

Values are n (%). *Relative risk (RR) and p values are from the chi-square test. MACE, Major Adverse Cardiovascular Event; MI, Myocardial Infarction; TLF, Target Lesion Failure; TLR, Target Lesion Revascularisation; and ST, Stent Thrombosis.

**Table 5:** Stent Thrombosis at 3 years.

Stent Thrombosis	Cordimax	Xience V	RR(95% CI)	*P* value
	(n = 2460)	(n = 1237)		
Definite	10 (0.4%)	7 (0.6%)	0.50 (0.07–3.57)	0.61
Probable	0 (0%)	0 (0%)	—	—
Definite or Probable	10 (0.4%)	7 (0.6%)	0.50 (0.07–3.57)	0.61

Values are n (%). *Relative risk (RR) and p values are from the chi-square test.

Univariate and multivariate analysis show that the location of the target lesion was an independent predictor of TLF following either Cordimax or Xience V implantation in patients (Table 6). Compared with the patients who developed lesions in other parts, patients with left main artery lesions exhibit a higher risk of the occurrence of TLF.

**Table 6:** Independent Predictors for Primary Endpoint (TLF) After Cordimax or Xience V Implantation in Overall Population.

Parameter	Univariate Analysis			Multivariate Analysis		
	HR	95% CI	*P* value	HR	95% CI	*P* value
Device	0.81	0.61–1.08	0.15	0.78	0.58–1.04	0.09
Gender	1.05	0.77–1.42	0.78	0.93	0.68–1.28	0.67
Hypertention	0.82	0.62–1.09	0.17	1.25	0.94–1.67	0.13
MI	0.87	0.50–1.53	0.64	1.11	0.63–1.95	0.72
Stroke	0.84	0.35–2.05	0.71	1.19	0.49–2.92	0.70
CCS			0.91			0.93
I	0.93	0.53–1.65	0.81	0.83	0.46–1.50	0.54
II	0.93	0.57–1.51	0.77	0.94	0.57–1.55	0.81
III	0.85	0.51–1.40	0.51	0.90	0.53–1.53	0.70
NYHA			0.29			0.27
I	0.83	0.26–2.63	0.75	0.81	0.25–2.62	0.72
II	0.65	0.21–2.07	0.47	0.60	0.18–1.95	0.40
III	0.60	0.18–1.96	0.40	0.60	0.18–2.01	0.41
Location of target lesion			0.00			<0.001
Left anterior descending coronary	0.34	0.20–0.59	<0.001	0.32	0.18–0.55	<0.001
Left circumflex coronary artery	0.31	0.17–0.58	<0.001	0.30	0.16–0.56	<0.001
Right coronary artery	0.33	0.19–0.58	<0.001	0.31	0.18–0.56	<0.001

CI, confidence interval; HR, hazard ratio.

## Discussion

The main purpose of this present work is to verify the efficacy and safety of the novel biodegradable polymer with an asymmetric coating, sirolimus-eluting stent (Cordimax) in relation to its long-term efficacy. Previously, Xience V stent from Abbott has been identified to be superior to the first-generation DES which, together with other favorable data, led to its approval by regulatory bodies^17^. Currently, clinical trials use Xience V as control group for their non-inferiority clinical trials^18^. Hence, the aim of the present study is to investigate the non-inferiority of clinical outcomes after implantation of the Cordimax stent when compared with the Xience V stent in patients undergoing coronary revascularization. This head-to-head comparison showed non-inferiority of the Cordimax biodegradable polymer sirolimus-eluting stent to the Xience V durable polymer everolimus-eluting stent in terms of both efficacy and safety. The cumulative rates for TLF at 1, 2 and 3 years were comparable for patients in the Cordimax and Xience V groups. However, the cumulative rate for TLF from 1 to 3 years in the Cordimax group was lower than that in the Xience V group (1.5% versus 3.1%, *P* < 0.001, respectively). These data hinted the tendency of the Cordimax stent to outperform the Xience V stent in long-term clinical benefits.

The rates for TLR in the Cordimax groups were low: 2.3% at 1 year and 3.6% at 3 years. These low rates were consistent with those reported in our previous trial on Cordimax^10^. In that randomized controlled trial the Cordimax stent proved to be non-inferior to the Cypher Select durable polymer sirolimus-eluting stent (Cordis Corporation, NJ, USA) for angiographic in-stent late luminal loss (LLL) at 9 month and MACE at 1year. The rates for CI-TLR for the Cordimax stent were 1.5% at 1 year and 3.0% at 5 years^10^. There was no definite and probable stent thrombosis (as defined by ARC criteria) in the Cordimax group while one case of definite very late stent thrombosis was seen in the Cypher Select group^10^. The cumulative rates for MACE, target vessel revascularization (TVR), cardiac death and MI at 1 and 5 years were comparable for the Cordimax and Cypher Select stents. However, the cumulative rates for MACE and TVR from 2 to 5 years in the Cordimax group were lower than those in the Cypher Select group^10^. Similar to present findings, these results implied the long-term clinical benefits of the Cordimax stent.

Meta-analyses reveal that new-generation DES are associated with lower rates of restenosis, stent thrombosis, and mortality compared with first-generation DES^19,20^. Xience V, a new-generation durable polymer everolimus‐eluting stent, was identified as being one of the most effective stents in reducing TVR/TLR^19^. In the RESOLUTE All Comers trial, ischemia driven TLR occurred in 5.1% of Xience V patients at 2 years^21^. This rate was numerically lower than the 2-year rates reported for the Taxus (5.9%)^22^, BioMatrix (6.3%)^23^ and Resolute (5.7%)^21^ stents. The clinical safety and efficacy of the Xience V stent were evaluated in the SPIRIT family of trials. In the larger SPIRIT III trial (n = 1,002), treatment with Xience V, as compared with Taxus, resulted in a significant 32% reduction in TVF (10.7% versus 15.4%, *P* = 0.04) and a 45% reduction in MACE (cardiac death, MI, or TLR; 7.3% versus 12.8%, *P* = 0.004) at a 2-year follow up^24^. The SPIRIT V trial for Xience V enrolled 2,700 patients with multiple de novo coronary artery lesions at 93 centers in Europe, Asia Pacific, Canada, and South Africa^25^. In this trial, MACE (all death, MI, and TLR) occurred in 2.7% of patients at 30 days. The rates of TLR and definite/probable stent thrombosis at 1 year were 1.8% and 0.66%, respectively. These data demonstrated the excellent safety and efficacy of the Xience V stent in a “real‐world” patient population. In the present study, low rates of TLF and MACE were observed in both Cordimax group and Xience V group, may associates with advances in coronary stent technology, and the inclusion of patients with the less complex coronary lesions. In addition, univariate and multivariate analysis show that the location of the target lesion was an independent predictor for TLF, patients with left main artery lesions exhibited higher rates of TLF, may associates with artery pressures caused by blood flow.

The continued presence of residual polymer after drug elution may cause chronic inflammation of the vessel wall, which is considered a potential trigger of stent thrombosis. This has motivated the development of DES with biodegradable polymers. Early randomized controlled trials showed that patients treated with biodegradable polymer stents had lower rates of stent thrombosis and subsequent TVR procedures than controls treated with first-generation DES^26^. However, the vast majority of trials comparing biodegradable polymer DES with new-generation durable polymer DES have only established non-inferiority. The Nobori® (Terumo, Japan) stents are biolimus-eluting stents with biodegradable polymer polylactic acid applied solely to the abluminal surface. In the NEXT trial^27^, the Nobori® biodegradable polymer biolimus-eluting stent showed non-inferiority but not superiority to the Xience V stents in terms of the primary efficacy endpoint of TLR at 1 year (4.2% versus 4.2%; *P* for non-inferiority < 0.0001; *P* for superiority = 0.93). In addition, the rate of definite stent thrombosis was low and comparable between the two groups (0.25% vs. 0.06%, *P* = 0.18).

The stent design, polymer coatings and the antiproliferative drug and its release kinetics have been implicated as mechanisms for delayed endothelial healing and chronic inflammation leading to stent thrombosis^5,6,28,29^. The Cordimax stent has a 316 L stainless steel platform, which is the gold standard material with a superior safety profile for stent application. Compared with a cobalt-based alloy, the 316 L stainless steel exhibits a much higher elasticity that allows better stent adhesion and helps reduce vascular damage at the site of implantation. Structurally, the Cordimax stent has a special “snap-fastener” design, in which the wave rod is connected with the S rod. This feature renders the Cordimax stent superior fatigue resistance. The Cordimax stent is coated solely at the abluminal stent site with polylactide-co-polyglycolide copolymer, which degrades into carbon dioxide and water after sirolimus is released^9^. Compared with conventionally coated stents, stents coated solely at the abluminal site tend to show reduced vascular restenosis and lower incidence of late stent thrombosis^9,10^. In addition, patented asymmetric coating and drug loading techniques are employed in the manufacture of the Cordimax stent to ensure favorable drug release^8^ and rapid vascular healing^9^. The better long-term outcomes seen with the Cordimax stent in this trial were in alignment with previous reports evaluating biodegradable polymer DES versus durable polymer DES^26^.

## Study Limitations

In this trial, the rates of TLF and MACE were low in both groups. This may be related to the inclusion of patients with relatively simple lesions (1–2 *in situ* coronary stenosis). Another limitation of this study was that we did not employ optical coherence tomography (OCT) or another imaging catheter to monitor the vascular wall condition at 1 month, 3 months and 6 months after the surgery. Finally, this study was limited to 3-year clinical follow-up. Ideally, 5-year follow-up results should be reported for a randomized trial of drug-eluting stents^30^.

## Conclusions

This study demonstrated non-inferiority of the Cordimax biodegradable polymer sirolimus-eluting stent to the Xience V durable polymer everolimus-eluting stent in terms of both efficacy and safety at 3 years.

## Methods

### Study design and patients

The study was a randomized, open-label, multicenter, non-inferiority trial. Patients older than 18 years of age who presented with symptomatic coronary artery disease or evidence of myocardial ischemia, 1–2 *in situ* coronary lesions, and at least one coronary lesion with ≥50% de novo stenosis in a vessel with a diameter of 2.5–4.0 mm between June, 2013 and May, 2014 were eligible for enrollment. The major exclusion criteria included a life expectancy of less than a year; pregnancy, known intolerance to the investigational device or concomitant medications (e.g., anticoagulants or antiplatelet drugs), anticipated discontinuation of dual antiplatelet therapy within the first 6 months, participation or anticipated participation in another clinical trial within 3 years, and uncertainty about the completion of 3-year follow-up. The trial was approved by the institutional ethics committee of Fudan University Affiliated Zhongshan Hospital and conducted in accordance with the provisions of the Declaration of Helsinki. This study is registered with ClinicalTrials.gov, number NCT03185221 (14/06/2017). All participating patients gave written informed consent.

### Sample size

According to the reported reference^13^, 2-year TLF of Xience V is about 8.3%. Assuming the TLF of Xience V and Cordimax groups are both 8.5%, the non-inferiority threshold is 3%, the power is 80%, one-sided inspection level is 0.025, with the lost of 20%, the estimated cases are 2440:1220 (2:1 allocation).

### Randomization

Patients were randomly assigned to receive either the Cordimax abluminal biodegradable polymer sirolimus-eluting stent (Rientech lnc., Shandong) or the Xience V durable polymer everolimus-eluting stent (Abbott Vascular, Redwood City, CA, USA) in a 2:1 allocation. A site stratified block randomization with randomly varying block sizes of 4 and 6 was performed. Random assignment was performed by a statistician from Fudan University and random envelopes were assigned to each site. Sequences were concealed from patients and clinical staff until assignment.

### Stents

The Cordimax sirolimus-eluting stent has a 316 L stainless steel platform coated on the abluminal surface with a biodegradable polylactide-co-polyglycolide copolymer (75:25 ratio) loaded with sirolimus^10^. The Cordimax stent was available in diameters of 2.5, 2.75, 3.0, 3.5 and 4.0 mm, and in lengths of 9, 12, 16, 18, 20, 23, 28 and 33 mm. The Xience V everolimus-eluting stent has a L-605 cobalt-chromium platform with a conformal coating of a non-erodible polymer loaded with everolimus^31^. The Xience V stent was available in diameters of 2.50, 2.75, 3.00, 3.5 and 4.00 mm, and in lengths of 8, 12, 15, 18, 23, and 28 mm.

### Study protocol

The stents were implanted using standard techniques. Patients received 300 mg aspirin and 300 mg clopidogrel at least 24 and six hours, respectively, before implantation, except for those who had been taking clopidogrel at 75 mg once daily for more than 72 hours. During the procedure, patients received unfractionated heparin at a dose of 100 IU/kg. Dual antiplatelet therapy with 100 mg aspirin and 75 mg clopidogrel once daily was continued for at least 1 year after stent implantation.

### Clinical endpoints and follow-up

The primary endpoint was the composite rate of target-lesion failure (TLF), defined as cardiac death, target-vessel MI, or clinically indicated target-lesion revascularization (TLR), at 1 years of stent implantation. The primary safety endpoint was a composite of death or myocardial infarction (MI) at 3 years. Secondary clinical safety and efficacy endpoints included major adverse cardiovascular events (MACE, defined as nonfatal MI, all-cause mortality, or TLR), the individual components of the composite endpoints, and definite or probable stent thrombosis, within 3 years of stent implantation. Cardiac death was defined as any death due to an evident cardiac cause, death related to the procedure, unwitnessed death, or death from unknown causes. A target-lesion revascularization was deemed clinically indicated if the stenosis of the treated lesion was at least 50% of the lumen diameter within the stent or within a 5-mm border on either side of the stent. Stent thrombosis (ST) was defined as the composite of any stent thromboses (definite, or probable) according to Academic Research Consortium (ARC) criteria^32^. Patients were systematically evaluated at 30 days, 6 months, 9 months, 12 months, 2 years, and 3 years by follow-up phone calls and office visits.

### Statistical analysis

The trial was powered to assess non-inferiority of the Cordimax sirolimus-eluting stent compared with the Xience V everolimus-eluting stent in terms of the primary endpoint (TLF) at 1 years. The published clinical trial results on the Xience V stent showed a TLF of 8.3% at 12 months^33^. On the basis of an assumed TLF of 8.5% in the Cordimax group, a prespecified non-inferiority margin of 0.03 (3%) with a one-sided type 1 error of 0.05, and a 2:1 (Cordimax/Xience V) ratio of patient allocation, the enrollment of 2034 patients in the Cordimax treatment arm and 1017 patients in the Xience V treatment arm was calculated to provide at least 80% power to detect non-inferiority^34^. Assuming a 20% loss-to-follow-up rate, a total sample size of 3660 patients (2440 in the Cordimax arm and 1220 in the Xience V arm) were required.

Analysis was performed on an intent-to-treat basis. Categorical variables are reported as numbers and percentages of patients, and continuous variables as means and SD. Treatment group comparisons were performed using the chi-square test or Fisher’s exact test for categorical outcomes and the Wilcoxon rank-sum test for continuous outcomes. Relative risk (RR) with 95% confidence interval (CI) and p values are reported. Non-inferiority was defined as the upper 95% confidence limit for the difference between treatment groups no greater than predetermined non-inferiority margin of 0.03 (3%). Further, to identify independent risk factors of TLF, univariate and multivariable Cox proportional hazard regression analyses were performed. A two-sided *P* value of less than 0.05 was considered to indicate statistical significance. Time-to-event analysis was performed using the Kaplan-Meier method, with treatment groups compared using the log-rank test. All analyses were performed with the SAS9.13 software (SAS Institute Inc., Cary, NC, USA).

## Data Availability

The datasets generated during and/or analyzed during the current study are available from the corresponding author on reasonable request.
